# Supplementary material for: Content-rich biological network constructed by mining PubMed abstracts
Source: BMC Bioinformatics. 2004 Oct 8;5:147. doi: 10.1186/1471-2105-5-147 (PMC528731; doi:10.1186/1471-2105-5-147)
Supplement: Additional File 5 — The original Chilibot query results of the term "long-term potentiation (LTP)" and 22 other terms, limiting the latest references analyzed to the years 1990, 1995, 2000, and 2004. [file 1471-2105-5-147-S5.bz2 › chilibotAdditionalFile5/ltp2004/html/SYNAPTOPHYSIN.html]

 


**SYNAPTOPHYSIN** (Input: SYNAPTOPHYSIN ) 

---


**Google Searches:** Entire Web  | EDU domain only  | PDF files only

.

**External Links:** OMIM |
LocusLink
| Swissprot
| GeneCards

  
**Maps of SYNAPTOPHYSIN**

Simple
Complete
graph in
radiant
tree
square
layout.

**New Hypothesis !**

**Synonyms** 

- synaptophysin   [PubMed]

**Synopsis**

- Our data show that synaptogyrin I and **synaptophysin** I perform redundant and essential functions in synaptic plasticity without being required for neurotransmitter release itself.  Neuron, 1999 
  [20]

- The data suggest a role for **synaptophysin** in release and indicate that activation of tyrosine kinase and **synaptophysin** phosphorylation contribute to long term potentiation  [LTP]  perhaps by modulating glutamate release.  Neuroreport, 1998 
  [20]

- In experiments in which cultured neurons were stained immunocytochemically with antibody against **synaptophysin** after electrophysiological recordings, hot spots were found to correspond to probable synaptic sites.  J Neurophysiol, 1995 
  [18]

- Decreased FM 1 43 staining correlated with low synapsin I and **synaptophysin** immunoreactivities, suggesting that decreased presynaptic function was commensurate with synaptic disassembly.  J Neurosci, 2002 
  [18]

- After training, pp60c src from hippocampus showed enhanced interactions with synaptic proteins such as synapsin I, **synaptophysin**, and the type 2 N methyl d aspartate receptor, as well as the cytoskeletal protein actin.  Proc Natl Acad Sci U S A, 2000 
  [16]

- We identify multiple accessory proteins associated with vesicle trafficking, including munc18, rSec8, DOC2, syntaxin 6, and **synaptophysin**, which have varied roles in regulated glutamate exocytosis.  Bone, 2001 
  [16]

- Dystrophicaxons in a 2 year old male Rottweiler with neuroaxonal dystrophy have shown **synaptophysin**, synapsin I, synaptosomal associated protein of 25 kDa SNAP 25, Rab 3a, and alpha synuclein immunoreactivity.  Acta Neuropathol (Berl), 2001 
  [16]

- Given the importance of syntaxin and **synaptophysin** in learning and memory processes and in the regulation of neurotransmitter release, changes in these proteins suggest their involvement in the associative learning aspects of sensitization and differential neurotransmitter release in the Nacc subregions.  Brain Res, 2001 
  [16]

- Analysis of another six mice from the same population that were showing weight loss and hypolocomotor activity exhibited a marked reactive gliosis as detected by immunoreactivity for glial fibrillary acidic protein and a profound loss of immunoreactivities for the presynaptic terminal vesicle marker proteins **synaptophysin** and synapsin and the dendritic marker microtubule associated protein 2 in many brain areas, but most predominantly in the cortex and hippocampus.  Neuroscience, 1999 
  [16]

- Since the number of functional release sites is approximately half the total number of **synaptophysin** immunoreactive sites, the PKA dependent activation of silent release sites of DG neuron terminals is suggested.  Neurosci Res, 2001 
  [16]

- Here, we show that late phase LTP L LTP is associated with a significant increase in numbers of synaptic puncta identified by **synaptophysin** and N cadherin, an adhesion protein involved in synapse formation during development.  Neuron, 2000 
  [16]

- Application of proteasome inhibitors caused accumulation of Vesl 1S immunoreactivity in the neurons which showed a punctate distribution in the cortical regions of the cells, and these puncta were found to be juxtaposed with **synaptophysin**, a presynaptic, synapse specific protein.  Brain Res Mol Brain Res, 2001 
  [15]

- The IP 3 R2 containing fine terminal branches of astrocyte processes in both brain regions were found juxtaposed to presynaptic terminals containing **synaptophysin** as well as PSD 95 containing postsynaptic densities.  Glia, 2002 
  [15]

- In addition, **synaptophysin**, synapsin I, and PSD 95 immunoreactivities were significantly reduced in both the gray and white matter of the LSSC during acute EAE episodes and EAE relapses, but showed partial recovery during EAE remission and after EAE recovery.  Am J Pathol, 2003 
  [15]

- Parallel increases in **synaptophysin** immunoreactivity were NOT observed, thus suggesting a selective induction of synapsin I after ischemia.  Brain Res, 1999 
  [15]
